# Apolipoproteins A1, B, and apoB/apoA1 ratio are associated with first ST-segment elevation myocardial infarction but not with recurrent events during long-term follow-up

**DOI:** 10.1007/s00392-018-1381-5

**Published:** 2018-10-08

**Authors:** Mathijs C. Bodde, Maaike P. J. Hermans, J. Wouter Jukema, Martin J. Schalij, Willem M. Lijfering, Frits R. Rosendaal, Fred P. H. T. M. Romijn, L. Renee Ruhaak, Arnoud van der Laarse, Christa M. Cobbaert

**Affiliations:** 1 Department of Cardiology, C5-P, Leiden University Medical Center, P.O. Box 9600, 2300 RC Leiden, The Netherlands; 2 Department of Epidemiology, Leiden University Medical Center, Leiden, The Netherlands; 3 Department of Clinical Chemistry and Laboratory Medicine, Leiden University Medical Center, Leiden, The Netherlands

**Keywords:** Apolipoproteins, Residual cardiovascular risk, Quantitative proteomics, STEMI

## Abstract

**Introduction:**

The current way to assess the risk of cardiovascular disease (CVD) is to measure conventional lipid and lipoprotein cholesterol fractions. Despite the success of statin treatment, residual cardiovascular risk remains high. Therefore, the value of extensive serum apolipoprotein (apo) profiling to assess the risk of ST-segment elevation myocardial infarction (STEMI) and of major adverse cardiac events (MACE) in patients with STEMI was investigated in a case–control design.

**Methods and results:**

Serum apo levels were measured using liquid chromatography and mass spectrometry in 299 healthy individuals and 220 patients with STEMI. First, the association of apo profiles in baseline samples with risk of STEMI was examined, and second, the association of apo profiles at baseline with risk of recurrent MACE in patients with STEMI in a longitudinal study design was studied. High baseline (> 1.25 g/L) apoA1 levels were associated with a decreased risk of STEMI [odds ratio (OR) 0.17; 95% CI 0.11–0.26], whereas high apoB (> 1.00 g/L) levels (OR 2.17; 95% CI 1.40–3.36) and apoB/apoA1 ratio (OR per 1 SD (OR/SD): 2.16; 95% CI 1.76–2.65) were associated with an increased risk. Very-low-density-lipoprotein (VLDL)-associated apos gave conflicting results. Neither conventional lipid levels nor apo levels were associated with MACE in the STEMI group.

**Conclusion:**

In conclusion, apoA1, apoB, and apoB/apoA1 were strongly associated with risk of STEMI. No clear relation between VLDL-associated apos and the risk of STEMI was found. Neither baseline serum apos nor lipids predicted MACE in statin-treated patients during long-term follow-up after a first STEMI.

## Introduction

Identification of patients at high risk for developing ST-segment elevation myocardial infarction (STEMI) is essential. Dyslipidemia in these patients is one of the factors that impair their long-term clinical outcome [1–4]. The current way to assess risk of cardiovascular disease (CVD) and its complications such as acute myocardial infarction (AMI), is, amongst others, quantification of total cholesterol (TC), LDL-cholesterol (LDLc), HDL-cholesterol (HDLc), and triglycerides (TG) concentrations in serum. Notwithstanding the success of statin treatment for reaching treatment goals, the residual cardiovascular risk being about 70% remains remarkably high [5–7]. The R3I initiative aims to explain this residual cardiovascular risk by looking for improved diagnostic, prognostic, and therapeutic biomarkers, beyond traditional risk factors [8].

The measurement of functional and structural protein components of lipoproteins, i.e., apolipoprotein (apos), is suggested to have additional value for coronary artery disease (CAD) risk assessment [9–15]. It has been suggested that apoB is a better marker of CAD risk than LDLc [9–11] and superior to non-HDLc [14, 16]. Furthermore, investigators from the INTERHEART study have shown that the apoB/apoA1 ratio was a better risk marker of AMI than the TC/HDLc ratio [13]. Recently, Pechlaner et al. presented in the Bruneck study new data about the relation of very-low-density-lipoprotein (VLDL)-associated apos, i.e., apoCII, apoCIII, and apoE, with incident CVD. These apos were found to be strong predictors of CVD [17]. The association of apoCIII with incident CVD was further corroborated by Van Capelleveen et al. [18] in a nested case–control study of the EPIC-Norfolk cohort. As residual cardiovascular risk remains high [8] even after successful treatment of traditional risk factors, a case–control study with long-term follow-up of patients with STEMI was executed to evaluate the value of extensive serum apo profiling for (1) prediction of STEMI and for (2) prediction of recurrent major adverse cardiac events (MACE) in patients with STEMI. The aim of this study was to focus on quantifying serum apoA1, apoB, apoCI, apoCII, apoCIII, and apoE, beyond serum lipids and lipoprotein cholesterol fractions. A previously developed method for quantitative serum apo profiling using liquid chromatography (LC) and mass spectrometry (MS), which has proven to be highly accurate and in concordance with quality requirements for medical tests, independent of the presence of hypertriglyceridemia, was used [19].

## Methods

### Study design

In the current study, the lipid and apo profiles in patients with STEMI from the MISSION! Intervention Trial [20] were compared with those of random digit dialing (RDD) controls from the Dutch Multiple Environmental and Genetic Assessment of risk factors for venous thrombosis (MEGA) study, a population-based study on risk factors for venous thrombosis [21]. Second, in patients with STEMI, the risk of recurrent MACE in a longitudinal study design was evaluated.

### Study participants

Patients with first STEMI admitted to the Leiden University Medical Center between February 2004 and October 2006 and included in the MISSION! Intervention Trial were included as cases. The study cohort consisted of 297 consecutive STEMI patients treated with primary percutaneous coronary intervention (pPCI). STEMI was defined as ongoing chest pain (> 30 min), accompanied with ST elevation (≥ 0.2 mV in ≥ 2 leads in V1–V3 or ≥ 0.1 mV in other leads) or presumed new left bundle branch block and a typical rise of high-sensitivity cardiac troponin-T (hs-cTnT). In case of out-of-hospital cardiac arrest, only patients with return of spontaneous circulation at the moment of arrival at the catheterization laboratory were included. Patients with prior AMI (*n* = 11), prior PCI (*n* = 3), and/or prior coronary artery bypass grafting (CABG) (*n* = 1) were excluded. A total of 5 patients had no available frozen serum samples for quantitative serum apo profiling. Since in the MEGA study, only controls were included who were < 70 years, MISSION patients who were > 70 years were excluded in this logistic regression analysis (*n* = 57). Therefore, in total, 220 cases were included. During the study, all patients were treated according to the institutional MISSION! protocol [22] based on guidelines of the European Society of Cardiology, American College of Cardiology, and the American Heart Association [23, 24]. The MISSION! protocol contains a standardized pre-hospital, in-hospital and outpatient clinical framework to optimize treatment. One of the in-hospital MISSION! performance indicators was to administrate statin therapy (rosuvastatin 10 mg) within 24 h after admission. In the outpatient phase, patients visited the outpatient clinic four times during the first year after STEMI. LDLc treatment goals at that time were < 2.5 mmol/L according to the guidelines at the time. Based on patients LDLc levels, statin therapy was adjusted according to reach the treatment goals [52, 53].

Controls were individuals who participated in the control arm of the MEGA study [19]. This case–control study included 4956 consecutive patients aged 18–70 years with a first deep vein thrombosis or pulmonary embolism, between March 1999 and September 2004. Partners of patients and individuals identified by RDD were asked to participate as controls. In total, 6297 controls (3297 partners and 3000 by random digit dialing) were included.

Of 3000 enrolled RDD controls, a random sample of 300 individuals was drawn for apo profile analysis and an extensive questionnaire including a list of potential risk factors for CVD. One RDD control was excluded because of a technical failure. No other exclusion criteria were applied to RDD controls.

### Data collection

Data of each MISSION! patient are systematically collected in the electronic patient file (EPD-VISION, Leiden) using a unique identification number. Of interest for the present analysis are the items on age, sex, and statin use at time of blood draw. Body mass index (BMI) was calculated by dividing weight (in kg) by height squared (m^2^). A BMI between 18.5 and 25 kg/m^2^ was defined as normal, between 25 and 30 kg/m^2^ as overweight, and ≥ 30 kg/m^2^ as obese. Patients using statins at the time of blood withdrawal were regarded as statin users.

### Follow-up in the MISSION! study

Information on all-cause mortality was obtained from the Dutch Municipality Records registry. Cause of death was retrieved from general practitioners. Clinical follow-up data were collected during the 30 days, 3-, 6-, and 12-month outpatient clinic visits. Follow-up data on serious adverse events including myocardial infarction, revascularisation, and stroke were obtained by telephone interviews at 2, 5, and 10 years after admission.

MACE was defined as the combined endpoint of 10-year clinical outcome, including death, AMI, revascularisation (PCI or CABG), and stroke.

### Blood collection and laboratory analysis

In the MISSION Intervention Trial, baseline blood samples were obtained at presentation (immediately before the PCI procedure was performed). The median time between onset of symptoms and blood withdrawal was 180 (IQR 120–252) min. Standard lab included non-fasting TC, HDLc, and TG, which were analysed directly. These levels enabled calculation of LDLc. An extra serum sample was coagulated for at least 60 min before centrifugation at 1500xg for 10 min at a temperature below 18 °C. Sera were pipetted into 1.1 mL Micronic tubes. Within 2 h after vena puncture, the serum samples were frozen in a − 70/− 80 °C freezer.

To determine the apo profile, a mass spectrometric method was developed for multiplexed quantification of six serum apos (apoA1, apoB, apoCI, apoCII, apoCIII, and apoE), including apoE phenotyping [19]. In contrast to classical HDLc and LDLc tests and lipoprotein particle counting methods, the quantitative proteomics test allows adequate quantification of unequivocally characterized apos with an analytical performance that meets test requirements derived from biological variation. Apo quantification by liquid chromatography (LC)–mass spectrometry (MS/MS) starts with solubilisation and denaturation of serum proteins before enzymatic digestion that generates signature peptides for the intact serum proteins. The peptides in the serum digest are separated by LC and detected by tandem MS/MS.

In MEGA controls, TC, TG, HDLc, apoA1, and apoB were measured on stored (− 80 °C) fasting serum samples. In 2015, apoA1 and apoB were measured with immunoturbidimetric tests on routine clinical chemistry analysers. In 2017, the complete apo profile (apoA1, apoB, apoCI, apoCII, apoCIII ,and apoE, plus apoE phenotyping) was measured in the same stored fasting serum samples that were thawed twice. Since apoA1 and apoB were measured twice, i.e., both in once and twice thawed sera, Bland–Altman plots and scatter plots were used to evaluate potential systematic error due to freeze-thawing once or twice. Results showed a mean difference of 0.09 g/L (95% CI − 0.12 to 0.30 g/L) for apoA1 and 0.08 g/L (95% CI − 0.08 to 0.23 g/L) for apoB, and *r*^2^ of 0.89 and 0.93, respectively. Since no systematic error appeared to be present, it was considered likely that freezing and thawing serum samples did not influence levels of apo profiles in MEGA.

Frozen storage of the serum samples was ensured by continuous, online temperature registration of the freezers.

TC and TG were measured by a colorimetric method (CHOD-PAP for TC and GPO-PAP for TG) on a Modular P analyser (Roche Diagnostics, Indianapolis, IN). HDLc was measured by a direct method based on the Kyowa Medex reaction principle using polyethylene glycol-modified enzymes (Roche Diagnostics, Indianapolis, IN). LDLc levels were calculated using the Friedewald formula [LDLc = TC − HDLc − (TG/2.2) for mmol/L]. If TG exceeded 4.52 mmol/L, LDLc was not calculated. In the MEGA study, the apo profile was determined similarly as in the MISSION! cohort. Remnant cholesterol concentration was calculated by TC − LDLc − HDLc.

### Cut-off points for lipid and apo profiles

The definitions of Nordestgaard et al. for abnormal lipid or apo levels in non-fasting state were used: TC 5.0 mmol/L, LDLc 3.0 mmol/L, HDLc 1.0 mmol/L, TG 2.0 mmol/L, remnant cholesterol 0.9 mmol/L, non-HDLc 3.9 mmol/L, apoA1 1.25 g/L, and apoB 1.0 g/L [25]. Since these cut-off points were not known for apoCI, apoCII, apoCIII, and apoE, quartile cut-off points of both conventional lipids and apos were used. One prior study analysed new apo profiles by a per standard deviation (SD) increase in controls,[17] which was also performed in the current study to evaluate whether this would yield similar results.

### Statistical analysis

Continuous variables with a normal distribution are presented as mean ± standard deviation (SD). Not-normally distributed data are presented as medians and interquartile range (IQR). The Mann–Whitney *U* test was used to test differences between two groups of not-normally distributed data. Lipid/apo profiles were log-transformed if not-normally distributed. Categorical variables are expressed as numbers and percentages.

To study determinants of conventional lipid and apo profiles in the general population, simple and multiple linear regression analyses were performed in the group of healthy individuals. To determine the association of various lipid and apo profiles with (1) risk of STEMI and (2) risk of MACE in patients with STEMI, odds ratios (ORs) with 95% confidence intervals (CIs) were calculated, and were adjusted for age, sex, and statin use by logistic regression methods. Model 1 in Table 3 is defined as the OR for the risk of STEMI adjusted for age, sex, and statin use. Model 1—statin users are the same model, but with statin users excluded.

Duration of follow-up was counted from time of first STEMI to end of follow-up, defined as 10-year follow-up, the date of a MACE, or lost to follow-up, whichever occurred first. Incidence rates of MACE or death were estimated as the number of events over the accumulated follow-up time. Cox-proportional hazards models were used to evaluate risks between groups, and were progressively adjusted for age, sex, and statin use. In all regression analyses, a preplanned sensitivity analysis was performed in which statin users were excluded at the time of blood draw (*n* = 40), as statin use do affect conventional lipid levels and apo profiles. To evaluate changes over time in lipid levels within patients a paired *T* test or a Wilcoxon signed rank test was performed when appropriate. All statistical tests were 2-tailed, *p* values < 0.05 were considered statistically significant. All statistical analyses were performed with SPSS for Windows, version 24.0 (SPSS Inc., IBM, Armonk, NY).

## Results

In total, 220 STEMI patients and 299 control subjects were eligible for this study. As expected, patients in the STEMI group were older (mean age 55.0 ± 9.37 years) than RDD controls (mean age 47.5 ± 13.1 years) in the control group. Patients with STEMI were more often men as compared with the control group (78.6% and 46.2%, respectively). Overall, the STEMI group had a lipid profile consistent with an increased cardiovascular risk. Whereas TC and LDLc levels did not differ between the two groups, HDLc was significantly lower and TG and remnant cholesterol were significantly higher in the STEMI group. ApoA1 was significantly lower in the STEMI group than in the control group (1.24 ± 0.24 g/L versus 1.53 ± 0.31 g/L, respectively, *p* < 0.001) and apoB was significantly higher in the STEMI group than in the control group (1.18 ± 0.25 g/L versus 1.08 ± 0.29 g/L, respectively, *p* < 0.001). All baseline characteristics are summarized in Table 1.

**Table 1:** Baseline characteristic table

	Patients (*n* = 220)	Controls (*n* = 299)	*p* value
Mean age, year (SD)	55.0 (9.37)	47.5 (13.1)	< 0.001
Men, *n* (%)	173 (78.6)	138 (46.2)	< 0.001
Statin use at blood draw, *n* (%)	18 (8.2)	22 (7.4)	0.717
Mean BMI, kg/m^2^ (SD)	26.63 (3.98)	25.32 (4.24)	< 0.001
Normal weight, *n* (%)	87 (40.1)	153 (52.4)	0.016
Overweight, *n* (%)	91 (41.9)	104 (35.6)	0.028
Obesity, *n* (%)	39 (18.0)	35 (12.0)	0.012
Smoking history, *n* (%)	139 (63.2)	174 (58.8)	0.312
Cholesterol, mmol/L, mean (SD)	5.63 (1.09)	5.54 (1.10)	0.333
Triglycerides, mmol/L, median (IQR)	1.53 (1.01–2.22)	1.23 (0.97–1.88)	0.002
HDLc, mmol/L, mean (SD)	1.27 (0.36)	1.37 (0.40)	0.004
LDLc, mmol/L, mean (SD)	3.48 (0.94)	3.49 (1.00)	0.912
Remnant cholesterol, mmol/L, mean (SD)	0.88 (0.72)	0.68 (0.36)	< 0.001
ApoA1, g/L, mean (SD)	1.24 (0.24)	1.53 (0.31)	0.001
ApoB, g/L, mean (SD)	1.18 (0.25)	1.08 (0.29)	< 0.001
ApoCI, mg/L, mean (SD)	20.03 (7.82)	21.38 (5.33)	0.020
ApoCII, mg/L, median (IQR)	36.10 (20.99–58.71)	34.32 (19.38–55.24)	0.440
ApoCIII, mg/L, median (IQR)	86.89 (65.48-117.08)	97.33 (77.56–119.90)	0.002
ApoE, mg/L, mean (SD)	33.15 (26.6)	30.71 (13.04)	0.170
Ratio apoB/apoA1, mean (SD)	1.00 (0.29)	0.74 (0.25)	0.001
Non-HDLc, mmol/L, mean (SD)	4.36 (1.07)	4.17 (1.13)	0.050

Categorical variables expressed by number (%)

Numerical variables expressed by mean (SD) or median (IQR)

Comparisons between groups were made using Chi square test for categorical variables and independent *T* test or Mann–Whitney *U* test for continuous variables

*BMI* body mass index, *HDLc* high-density-lipoprotein cholesterol, *LDLc* low-density-lipoprotein cholesterol, *Apo* apolipoprotein

Table 2 shows the association of conventional lipids and apos with cardiovascular risk factors (age, gender, smoking, obesity, and statin use) in control subjects. In these individuals, older age was correlated with significantly higher levels of almost all lipid and apo levels. Female controls had a slightly more favorable apo profile with a significantly higher apoA1 and lower apoB, than male controls. Furthermore, smoking and obesity were associated with significantly higher levels of apoB, but not with significantly higher LDLc levels.

**Table 2:** Association of lipid and apo profiles with cardiovascular risk factors in control subjects

	Age		Sex		Smoking history		Obesity		Statin use	
	< 50 y	≥ 50 years	Men	Women	No	Yes	No	Yes	No	Yes
Cholesterol										
Mean level, mmol/L	5.22	5.93	5.59	5.50	5.34	5.68	5.54	5.64	5.56	5.22
Mean difference (95% CI)	Ref	0.71 (0.47–0.95)***	Ref	− 0.09 (− 0.34 to 0.17)¯	Ref	0.33 (0.08–0.59)**	Ref	0.11 (− 0.28 to 0.50)¯	Ref	− 0.34 (− 0.82 to 0.14)¯
Mean difference (95% CI)*	Ref	0.77 (0.52–1.02)***	Ref	− 0.05 (− 0.29 to 0.19)¯	Ref	0.22 (− 0.03 to 0.47)¯	Ref	0.13 (− 0.24 to 0.50)¯	Ref	− 0.69 (− 1.18 to − 0.20)**
Mean difference(95% CI)†	Ref	0.79 (0.54–1.04)***	Ref	− 0.08 (− 0.33 to 0.16)¯	Ref	0.20 (− 0.05 to 0.48)¯	Ref	0.14 (− 0.25 to − 0.52)¯	NA	
Triglycerides, mmol/L										
Mean level, mmol/L	1.41	1.63	1.60	1.44	1.37	1.60	1.49	1.68	1.50	1.70
Mean difference (95% CI)	Ref	0.21 (0.03–0.40)**	Ref	− 0.15 (− 0.34 to 0.03)¯	Ref	0.23 (0.04–0.42)**	Ref	0.19 (− 0.10 to 0.48)¯	Ref	0.19 (− 0.16 to 0.55)¯
Mean difference (95% CI)*	Ref	0.14 (− 0.06 to 0.33)¯	Ref	− 0.13 (− 0.32 to 0.06)¯	Ref	0.21 (0.02–0.40)**	Ref	0.21 (− 0.08 to 0.49)¯	Ref	0.12 (− 0.26 to 0.051)¯
Mean difference(95% CI)†	Ref	0.15 (− 0.05 to 0.35)¯	Ref	− 0.14 (− 0.34 to 0.06)¯	Ref	0.17 (− 0.03 to 0.38)¯	Ref	0.23 (− 0.08 to 0.54)¯	NA	
HDLc, mmol/L										
Mean level, mmol/L	1.34	1.41	1.23	1.49	1.42	1.34	1.39	1.22	1.37	1.26
Mean difference (95% CI)	Ref	0.07 (− 0.02 to 0.16)¯	Ref	0.26 (0.17–0.35)***	Ref	− 0.08 (− 0.17 to 0.02)¯	Ref	− 0.17 (− 0.31 to − 0.03)*	Ref	− 0.09 (− 0.26− 0.08)¯
Mean difference (95% CI)*	Ref	0.13 (0.04–0.22)**	Ref	0.27 (0.18–0.35)***	Ref	− 0.07 (− 0.16 to 0.02)¯	Ref	− 0.20 (− 0.34 to − 0.07)*	Ref	− 0.09 (− 0.26 to 0.09)¯
Mean difference(95% CI)†	Ref	0.12 (0.03–0.22)**	Ref	0.26 (0.17–0.35)***	Ref	− 0.05 (− 0.15 to 0.04)¯	Ref	− 0.19 (− 0.33 to − 0.05)*	NA	
LDLc, mmol/L										
Mean level, mmol/L	3.24	3.79	3.64	3.36	3.31	3.61	3.47	3.67	3.51	3.18
Mean difference (95% CI)	Ref	0.55 (0.33–0.76)***	Ref	− 0.27 (− 0.50 to − 0.05)**	Ref	0.30 (0.07–0.53)**	Ref	0.19 (− 0.16 to 0.55)¯	Ref	− 0.34 (− 0.77 to 0.10)¯
Mean difference (95% CI)*	Ref	0.58 (0.35–0.81)***	Ref	− 0.26 (− 0.48 to − 0.04)**	Ref	0.20 (− 0.03 to 0.42)¯	Ref	0.24 (− 0.10 to 0.58)¯	Ref	− 0.66 (− 1.11 to − 0.22)¯
Mean difference(95% CI)†	Ref	0.59 (0.37–0.82)***	Ref	− 0.28 (− 0.50 to − 0.06)**	Ref	0.18 (− 0.05 to 0.40)¯	Ref	0.22 (− 0.13 to 0.58)¯	NA	
Remnant cholesterol										
Mean level, mmol/L	0.64	0.73	0.72	0.65	0.62	0.72	0.67	0.76	0.68	0.76
Mean difference (95% CI)	Ref	0.10 (0.01–0.18)**	Ref	0.07 (− 0.15 to 0.01)¯	Ref	0.10 (0.02–0.19)**	Ref	0.09 (− 0.04 to 0.22)¯	Ref	0.09 (− 0.07 to 0.25)¯
Mean difference (95% CI)*	Ref	0.06 (− 0.03 to 0.15)¯	Ref	− 0.06 (− 0.14 to 0.03)¯	Ref	0.09 (0.01–0.18)**	Ref	0.09 (− 0.04 to 0.22)¯	Ref	0.06 (− 0.12 to 0.23)¯
Mean difference(95% CI)†	Ref	0.07 (− 0.02 to 0.16)¯	Ref	− 0.06 (− 0.15 to 0.03)¯	Ref	0.08 (− 0.01 to 0.17)¯	Ref	0.10 (− 0.04 to 0.25)¯	NA	
Apo A1, g/L										
Mean level	1.50	1.58	1.42	1.63	1.56	1.52	1.54	1.49	1.54	1.48
Mean difference (95% CI)	Ref	0.08 (0.01–0.15)**	Ref	0.21 (0.14–0.28)***	Ref	− 0.04 (− 0.11 to 0.03)¯	Ref	0.05 (− 0.16 to 0.06)¯	Ref	− 0.06 (− 0.19 to 0.08)¯
Mean difference (95% CI)*	Ref	0.12 (0.05–0.19)***	Ref	0.21 (0.14–0.28)***	Ref	− 0.04 (− 0.11 to 0.03)¯	Ref	− 0.07 (− 0.17 to 0.03)¯	Ref	− 0.07 (− 0.20 to 0.07)¯
Mean difference(95% CI)†	Ref	0.12 (0.05–0.19)***	Ref	0.20 (0.13–0.27)***	Ref	− 0.02 (− 0.10 to 0.05)¯	Ref	− 0.06 (− 0.17 to 0.05)¯	NA	
Apo B, g/L										
Mean level	1.02	1.16	1.14	1.04	1.02	1.13	1.07	1.19	1.09	1.06
Mean difference (95% CI)	Ref	0.14 (0.08–0.21)***	Ref	− 0.09 (− 0.16 to − 0.03)**	Ref	0.11 (0.04–0.17)**	Ref	0.12 (0.01–0.22)**	Ref	− 0.02 (− 0.15 to 0.10)¯
Mean difference (95% CI)*	Ref	0.13 (0.07–0.20)***	Ref	− 0.09 (− 0.15 to − 0.03)**	Ref	0.08 (0.02–0.15)**	Ref	0.13 (0.03–0.23)**	Ref	− 0.11 (− 0.24 to 0.02)¯
Mean difference(95% CI)†	Ref	0.14 (0.07–0.21)***	Ref	− 0.10 (− 0.16 to − 0.03)**	Ref	0.12 (0.02–0.23)**	Ref	0.07 (0.00–0.14)**	NA	
Apo C-I, mg/L										
Mean level	20.23	22.77	21.30	21.44	20.80	21.78	21.35	21.40	21.37	21.53
Mean difference (95% CI)	Ref	2.54 (1.35–3.72)***	Ref	0.14 (− 1.08 to 1.36)¯	Ref	0.98 (− 0.27 to 2.22)¯	Ref	0.05 (− 1.85 to 1.96)¯	Ref	0.16 (− 2.17 to 2.49)¯
Mean difference (95% CI)*	Ref	2.65 (1.36–3.93)***	Ref	0.26 (− 0.98 to 1.49)¯	Ref	0.52 (− 0.74 to 1.78)¯	Ref	− 0.04 (− 1.92 to 1.85)¯	Ref	− 1.02 (− 3.51 to 1.48)¯
Mean difference(95% CI)†	Ref	2.70 (1.40–4.01)***	Ref	0.00 (− 1.28 to 1.28)¯	Ref	0.12 (− 1.90 to 2.13)¯	Ref	0.61 (− 0.70 to 1.92)¯	NA	
Apo C-II, mg/L										
Mean level	31.71	49.85	45.52	35.08	35.98	42.13	38.65	47.04	38.80	53.68
Mean difference (95% CI)	Ref	18.14 (12.15–24.12)***	Ref	− 10.44 (− 16.65 to − 4.23)***	Ref	6.16 (− 0.23 to 12.54)¯	Ref	8.39 (− 1.38 to 18.16)¯	Ref	15.88 (2.92–26.83)**
Mean difference (95% CI)*	Ref	15.31 (8.99–21.63)***	Ref	− 9.19 (− 15.27 to − 3.10)**	Ref	2.80 (− 3.42 to 9.01)¯	Ref	7.78 (− 1.51 to 17.07)¯	Ref	5.02 (− 7.28 to 17.33)¯
Mean difference(95% CI)†	Ref	15.39 (8.94–21.84)***	Ref	− 9.89 (− 16.22 to − 3.56)**	Ref	9.54 (− 0.42 to 19.49)¯	Ref	2.41 (− 4.07 to 8.88)¯	NA	
Apo C-III, mg/L										
Mean level	95.77	113.07	102.77	104.28	99.73	105.93	103.20	107.10	102.51	117.17
Mean difference (95% CI)	Ref	17.30 (9.03–25.57)**	Ref	1.51 (− 6.98 to 10.00)¯	Ref	6.20 (− 2.41 to 14.82)¯	Ref	3.89 (− 9.37 to 17.16)¯	Ref	14.67 (− 1.45 to 30.79)¯
Mean difference (95% CI)*	Ref	14.90 (5.98–23.82)**	Ref	3.80 (− 4.79 to 12.38)¯	Ref	4.69 (− 4.08 to 13.45)¯	Ref	2.42 (− 10.67 to 15.52)¯	Ref	9.02 (− 8.33 to 26.40)¯
Mean difference(95% CI)†	Ref	14.56 (5.36–23.77)**	Ref	3.23 (− 5.81 to 12.26)¯	Ref	4.68 (− 9.53 to 18.89)¯	Ref	5.61 (− 3.62 to 14.89)¯	NA	
Apo E, mg/L										
Mean level	27.91	34.12	30.46	30.93	30.40	30.74	30.37	32.20	30.69	30.06
Mean difference (95% CI)	Ref	6.21 (3.31–9.11)***	Ref	0.46 (− 2.52 to 3.44)¯	Ref	0.37 (− 2.66 to 3.40)¯	Ref	1.87 (− 2.59 to 6.32)¯	Ref	0.37 (− 5.32 to 6.06)¯
Mean difference (95% CI)*	Ref	5.96 (2.96–8.95)***	Ref	0.46 (− 2.42 to 3.35)¯	Ref	− 0.65 (− 3.60 to 2.29)¯	Ref	1.60 (− 2.80 to 6.00)¯	Ref	− 2.47 (− 8.30 to 3.36)¯
Mean difference(95% CI)†	Ref	5.71 (2.75–8.68)***	Ref	− 0.28 (− 3.18 to 2.63)¯	Ref	2.93 (− 1.65 to 7.50)¯	Ref	− 0.10 (− 3.07 to 2.87)¯	NA	
Ratio ApoB/ApoA1										
Mean level, mmol/L	0.71	0.76	0.82	0.67	0.68	0.78	0.73	0.82	0.74	0.74
Mean difference (95% CI)	Ref	0.05 (− 0.01 to 0.11)¯	Ref	− 0.15 (− 0.21 to − 0.10)***	Ref	0.10 (0.04–0.16)***	Ref	0.09 (0.00–0.18)**	Ref	0.06 (− 0.11 to 0.12)¯
Mean difference (95% CI)*	Ref	0.03 (− 0.03 to 0.08)¯	Ref	− 0.15 (− 0.21 to − 0.09)***	Ref	0.09 (0.03–0.14)**	Ref	0.11 (0.03–0.20)**	Ref	− 0.04 (− 0.15 to 0.08)¯
Mean difference(95% CI)†	Ref	0.03 (− 0.03 to 0.09)¯	Ref	− 0.15 (− 0.21 to − 0.09)***	Ref	0.07 (0.01–0.13)**	Ref	0.11 (0.02–0.20)**	NA	
Non-HDLc, mmol/L										
Mean level, mmol/L	3.88	4.52	4.36	4.01	3.93	4.33	4.14	4.42	4.19	3.94
Mean difference (95% CI)	Ref	0.64 (0.39–0.89)***	Ref	− 0.35 (− 0.60 to − 0.09)**	Ref	0.41 (0.15–0.67)**	Ref	0.28 (− 0.12 to 0.68)¯	Ref	− 0.25 (− 0.74 to 0.24)¯
Mean difference (95% CI)*	Ref	0.64 (0.38–0.90)***	Ref	− 0.32 (− 0.57 to − 0.07)**	Ref	0.29 (0.04–0.55)**	Ref	0.33 (− 0.05 to 0.71)¯	Ref	− 0.61 (− 1.11 to − 0.10)**
Mean difference(95% CI)†	Ref	0.66 (0.40–0.92)***	Ref	− 0.34 (− 0.60 to − 0.09)**	Ref	0.25 (− 0.01 to 0.52)¯	Ref	0.33 (− 0.08 to 0.73)¯	NA	

Significance: ***p ≤ 0.001, **p ≤ 0.05, ¯*p* > 0.05

*Ref* reference, *NA* not applicable

*Multivariate adjusted for age, sex, smoking status, obesity and statin use

†Multivariate adjusted for age, sex, smoking status and obesity, statin users excluded

**Fig. 1:**
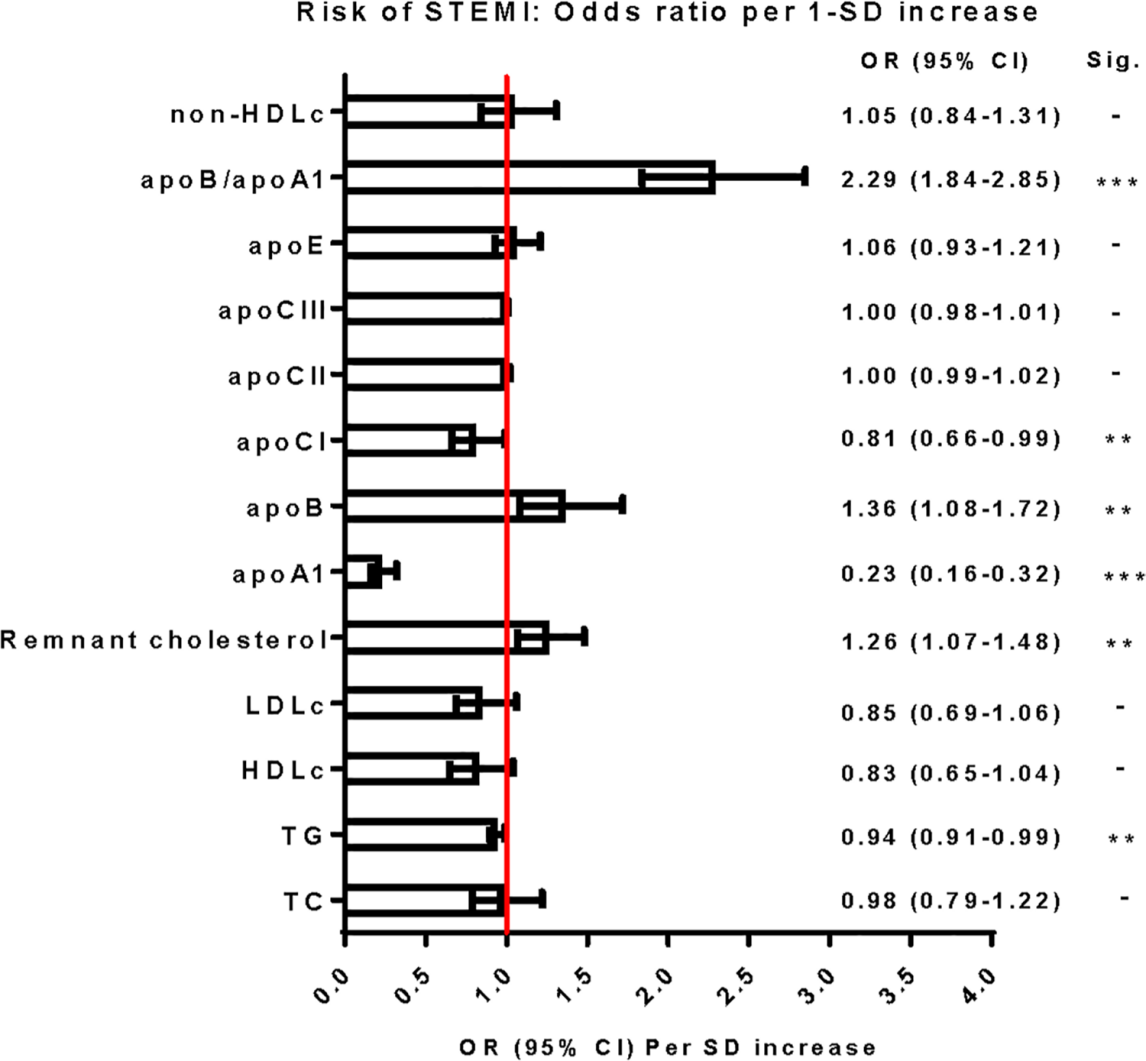
Odds ratio for a 1-SD higher (95% CI) risk of STEMI for each individual lipid marker. The risk was adjusted for age and sex. Statin users were excluded. Significance: ***p < 0.001, **p < 0.05, -*p* > 0.05. 1 SD corresponds to: TC, 1.10 mmol/L; TG, 0.81 mmol/L; HDLc, 0.40 mmol/L; LDLc, 1.00 mmol/L; remnant cholesterol, 0.36 mmol/L; apoA1, 0.31 g/L; apoB, 0.29 g/L; apoCI, 5.33 mg/L; apoCII, 27.64 mg/L; apoCIII, 37.12 mg/L; apoE, 13.04 mg/L; apoB/apoA1, 0.25; non-HDLc, 1.13 mmol/L. *Apo* apolipoprotein, *OR* Odds Ratio, *STEMI* ST-segment elevation myocardial infarction, *SD* standard deviation, *TC* total cholesterol, *TG* triglycerides, *HDLc* high-density-lipoprotein cholesterol, *LDLC* low-density-lipoprotein cholesterol, *Apo* apolipoprotein

Table 3 demonstrates the OR of STEMI for various lipid and apo profiles. These are shown as high versus low levels, divided per quartile and per 1-SD increase. ORs were adjusted for age, gender, and statin use (model 1). Figure 1 illustrates the association of risk of STEMI per 1-SD increase for each lipid or lipoprotein, in which statin users are excluded (Model 1—statin users). TC and LDLc showed no association with the risk of STEMI. The adjusted OR for high HDLc was 0.51 (95% CI 0.32–0.82) which was similar in the group without statin users. High remnant cholesterol levels were significantly associated with risk of STEMI with an OR of 1.61 (95% CI 1.03–2.51). The OR per SD increase (0.36 mmol/L) was 1.21 (95% CI 1.03–2.42).

**Table 3:** Risk of STEMI according various levels of lipoproteins and apolipoproteins

	Level		Cases	Controls	Univariate analysis		Model 1			Model 1-statin users	
					Odds ratio	(95%)	Adjusted odds ratio *†*	(95% CI)		Adjusted odds ratio#	(95% CI)
Total-c, mmol/L											
High		> 5.0	159 (72)	202 (68)	1.25¯	(0.85–1.83)	0.88¯	(0.56–1.38)		0.85¯	(0.52–1.38)
Quartiles	1	< 4.82	45 (21)	75 (25)	1	(reference)	1	(reference)		1	(reference)
	2	4.82–5.52	65 (30)	74 (25)	1.46¯	(0.89–2.41)	1.23¯	(0.69–2.17)		1.29¯	(0.69–2.40)
	3	5.52–6.20	49 (22)	75 (25)	1.09¯	(0.65–1.82)	0.74¯	(0.41–1.35)		0.81¯	(0.43–1.52)
	4	≥ 6.20	61 (28)	75 (25)	1.36¯	(0.82–2.24)	0.90¯	(0.54–1.73)		1.07¯	(0.57–2.01)
Per SD increase		1.10			1.09 ¯	(0.92–1.30)	0.94¯	(0.7–1.16)		0.98¯	(0.79–1.22)
Triglycerides, mmol/L											
High		> 2.0	65 (30)	60 (20)	1.67**	(1.11–2.05)	1.42¯	(0.92–2.21)		1.60**	(1.00-2.56)
Quartiles	1	< 0.97	44 (20)	70 (23)	1	(reference)	1	(reference)		1	(reference)
	2	0.97–1.23	34 (16)	79 (26)	0.69¯	(0.40–1.19)	0.59¯	(0.32–1.09)		0.53¯	(0.27–1.01)
	3	1.23–1.88	64 (29)	75 (25)	1.36¯	(0.82–2.25)	0.93¯	(0.53–1.63)		0.99¯	(0.54–1.79)
	4	> 1.88	78 (36)	75 (25)	1.66¯	(1.01–2.71)	1.13¯	(0.66–1.95)		1.21¯	(0.68–2.16)
Per ln SD increase		0.81			0.93***	(0.90–0.97)	0.95**	(0.91–0.99)		0.94**	(0.91–0.99)
HDLc, mmol/L											
High		> 1.0	159 (72)	257 (86)	0.43***	0.27–0.66	0.51**	(0.32–0.82)		0.47**	(0.28–0.78)
Quartiles	1	< 1.10	72 (33)	74 (25)	1	(reference)	1	(reference)		1	(reference)
	2	1.10–1.29	50 (23)	74 (25)	0.69-	(0.43–1.13)	0.72¯	(0.42–1.22)		0.77¯	(0.45–1.35)
	3	1.29–1.56	52 (24)	73 (24)	0.73-	(0.45–1.19)	0.88¯	(0.51–1.51)		0.84¯	(0.47–1.48)
	4	> 1.56	46 (21)	78 (26)	0.61**	(0.37–0.99)	0.81¯	(0.46–1.43)		0.73¯	(0.40–1.33)
Per SD increase		0.40			0.76**	(0.63–0.92)	0.85¯	(0.67–1.06)		0.83¯	(0.65–1.04)
LDLc, mmol/L											
High		> 3.0	156 (71)	196 (66)	1.28¯	(0.88–1.87)	0.86¯	(0.55–1.34)		0.89¯	(0.55–1.43)
Quartiles	1	< 2.77	47 (21)	74 (25)	1	(reference)	1	(reference)		1	(reference)
	2	2.77–3.47	56 (26)	76 (25)	1.16¯	(0.70–1.92)	0.85¯	(0.47–1.53)		0.89¯	(0.47–1.67)
	3	3.47–4.12	71 (32)	74 (25)	1.51¯	(0.93–2.47)	1.05¯	(0.59–1.86)		1.08¯	(0.59–2.01)
	4	> 4.12	46 (21)	75 (25)	0.97¯	(0.58–1.62)	0.58¯	(0.32–1.08)		0.63¯	(0.33–1.20)
Per SD increase		1.00			0.99¯	(0.83–1.19)	0.82¯	(0.66–1.01)		0.85¯	(0.69–1.06)
Remnant cholesterol, mmol/L											
High		0.9	75 (34)	60 (20)	2.06***	(1.39–3.07)	1.72**	(1.12–2.65)	1.92**		(1.22–3.05)
Quartiles	1	< 0.44	44 (20)	75 (25)	1	(reference)	1	Reference	1		Reference
	2	0.44–0.56	33 (15)	74 (25)	0.76¯	(0.44–1.32)	0.67¯	(0.36–1.24)	0.62¯		(0.33–1.19)
	3	0.56–0.85	63 (29)	76 (25)	1.41¯	(0.86–2.33)	0.98¯	(0.56–1.71)	1.05¯		(0.59–1.89)
	4	0.85	80 (36)	74 (25)	1.84¯	(1.13-3.00)	1.28¯	(0.75–2.19)	1.37¯		(0.78–2.42)
Per SD increase		0.36			1.35***	(1.17–1.57)	1.25**	(1.07–1.45)		1.26**	(1.07–1.48)
Apo A1, g/L											
High		> 1.25	96 (42)	246 (82)	0.17***	(0.11–0.25)	0.17***	(0.11–0.26)		0.15	(0.09–0.25)
Quartiles	1	< 1.31	153 (70)	75 (25)	1	(reference)	1	(reference)		1	(reference)
	2	1.31–1.48	30 (14)	73 (24)	0.20***	(0.12–0.33)	0.18***	(0.10–0.32)		0.15***	(0.09–0.24)
	3	1.48–1.72	28 (13)	74 (25)	0.19***	(0.11–0.31)	0.15***	(0.08–0.27)		0.15***	(0.08–0.27)
	4	> 1.72	9 (4)	77 (26)	0.06***	(0.03–0.12)	0.06***	(0.02–0.13)		0.05***	(0.02–0.12)
Per SD increase		0.31			0.26***	(0.20–0.34)	0.24***	(0.18–0.33)		0.23***	(0.16–0.32)
Apo B, g/L											
High		> 1.0	172 (78)	168 (56)	2.79***	(1.89–4.14)	2.17***	(1.40–3.36)		2.46***	(1.53–3.95)
Quartiles	1	< 0.87	17 (8)	74 (25)	1	(reference)	1	(reference)		1	(reference)
	2	0.87–1.04	43 (20)	72 (24)	2.60**	(1.36–4.97)	1.82¯	(0.89–3.72)		3.05**	(1.29–7.23)
	3	1.04–1.26	74 (34)	77 (26)	4.18***	(2.26–7.75)	2.89**	(1.47–5.68)		4.67**	(2.06–10.63)
	4	> 1.26	86 (39)	76 (25)	4.93***	(2.67–9.07)	3.04**	(1.55–5.95)		5.11***	(2.27–11.53)
Per SD increase		0.29			1.48***	(1.22–1.80)	1.27**	(1.02–1.57)		1.36**	(1.08–1.72)
Apo C-I, mg/L											
Quartiles	1	< 17.40	85 (39)	74 (25)	1	(reference)	1	(reference)		1	(reference)
	2	17.40–20.80	54 (25)	75(25)	0.63¯	(0.39-1.00)	0.50**	(0.29–0.85)		0.48**	(0.27–0.85)
	3	20.80-24.74	44 (20)	76 (25)	0.50**	(0.31–0.82)	0.35***	(0.20–0.60)		0.40**	(0.22–0.71)
	4	> 24.74	37 (17)	74 (25)	0.44***	(0.26–0.72)	0.32***	(0.18–0.57)		0.37***	(0.20–0.68)
Per SD increase		5.33			0.82**	(0.69–0.97)	0.76**	(0.62–0.92)		0.81**	(0.66–0.99)
Apo C-II, mg/L											
Quartiles	1	< 19.38	47 (21)	74 (25)	1	(reference)	1	(reference)		1	(reference)
	2	19.38–34.32	58 (26)	75 (25)	1.22¯	(0.74–2.01)	0.83¯	(0.46–1.47)		0.90¯	(0.50–1.65)
	3	34.32–55.24	52 (24)	76 (25)	1.08¯	(0.65–1.79)	0.62¯	(0.35–1.12)		0.73¯	(0.39–1.35)
	4	> 55.24	63 (29)	74 (25)	1.34¯	(0.82–2.20)	0.64¯	(0.36–1.15)		0.80¯	(0.43–1.48)
Per ln SD increase		27.64			1.01¯	(1.00-1.03)	1.00¯	(0.98–1.02)		1.00¯	(0.99–1.02)
Apo C-III, mg/L											
Quartiles	1	< 77.56	81 (37)	74 (25)	1	(reference)	1	(reference)		1	(reference)
	2	77.56–97.33	51 (23)	75 (25)	0.62**	(0.39-1.00)	0.54***	(0.32–0.93)		0.53**	(0.30–0.94)
	3	97.33–119.90	40 (18)	76 (25)	0.48**	(0.29–0.79)	0.32***	(0.18–0.56)		0.37***	(0.21–0.67)
	4	> 119.90	48 (22)	74 (25)	0.59**	(0.37–0.96)	0.41**	(0.24–0.72)		0.51**	(0.28–0.91)
Per ln SD increase		37.12			1.00¯	(0.98–1.01)	0.99¯	(0.98–1.01)		1.00¯	(0.98–1.01)
Apo E, mg/L											
Quartiles	1	< 22.14	45 (21)	75 (25)	1	(reference)	1	(reference)		1	(reference)
	2	22.14–28.19	61 (28)	75 (25)	1.36¯	(0.82–2.24)	1.10¯	(0.62–1.95)		1.26¯	(0.68–2.32)
	3	28.19–36.55	62 (28)	74 (25)	1.40¯	(0.85–2.30)	0.98¯	(0.55–1.73)		1.02¯	(0.55–1.88)
	4	> 36.55	52 (24)	75 (25)	1.16¯	(0.69–1.93)	0.86¯	(0.48–1.54)		0.99¯	(0.53–1.86)
Per SD increase		13.04			1.09¯	(0.95–1.26)	1.04¯	(0.93–1.18)		1.06¯	(0.93–1.21)
Ratio ApoB/ApoA1											
Quartiles	1	< 0.53	5 (2)	74 (25)	1	(reference)	1	(reference)		1	(reference)
	2	0.53–0.70	27 (12)	78 (26)	5.12***	(1.87–14.01)	3.52**	(1.24–10.03)		2.88¯	(0.89–9.30)
	3	0.70–0.88	50 (23)	70 (23)	10.57***	(3.99–28.05)	6.99***	(2.52–19.39)		7.21***	(2.33–22.34)
	4	> 0.88	138 (63)	77 (26)	26.53***	(10.28–68.42)	16.20***	(5.97–43.91)		16.98***	(5.67–50.85)
Per SD increase		0.25			2.39***	(1.98–2.89)	2.16***	(1.76–2.65)		2.29***	(1.84–2.85)
Non-HDLc, mmol/L											
Quartiles	1	< 3.32	33 (15)	73 (24)	1	(reference)	1	(reference)		1	(reference)
	2	3.32–4.07	47 (21)	77 (26)	1.35¯	(0.78–2.34)	0.92¯	(0.49–1.72)		0.75¯	(0.56–2.24)
	3	4.07–4.96	85 (39)	74 (25)	2.54***	(1.52–4.26)	1.74¯	(0.96–3.14)		1.94**	(1.01–3.72)
	4	> 4.96	55 (25)	75 (25)	1.62¯	(0.95–2.78)	1.04¯	(0.54–1.86)		1.21¯	(0.62–2.37)
Per SD increase		1.13			1.20¯	(1.00-1.43)	1.00¯	(0.81–1.22)		1.05¯	(0.84–1.31)

Significance: ***p ≤ 0.001; **p ≤ 0.05; ¯*p* > 0.05

#Model 1-statin users; adjusted for age and sex, statin users excluded

†Model 1; adjusted for age, sex and statin use

High apoA1 levels were strongly associated with risk of STEMI with an OR of 0.17 (95% CI 0.11–0.26). Per SD increase of apoA1 (0.31 g/L) the OR was 0.24 (95% CI 0.18–0.33). These results were similar in the group without statin users. High apoB levels were also associated with risk of STEMI with an OR of 2.17 (95% CI 1.40–3.36). This effect appeared to be even stronger in the group without statin users (OR 2.46; 95% CI 1.53–3.95). Per SD increase of apoB (0.29 g/L), the OR of STEMI was 1.27 (95% CI 1.02–1.57) in the total group, and 1.36 (95% CI 1.08–1.72) in the group without statin users. High baseline apoB/apoA1 ratios were also associated with STEMI risk. The OR per SD increase (0.25) was 2.16 (95% CI 1.76–2.65) in the total group and 2.29 (95% CI 1.84–2.85) in the group without statin users.

High apoCI levels were associated with risk of STEMI with an OR of 0.32 (95% CI 0.18–0.57) of the highest quartile versus the lowest quartile. Per SD increase of apoCI (5.33 mg/L), the OR was 0.76 (95% CI 0.62–0.93). The VLDL-associated apos gave conflicting results. No association of apoCII and apoE with the risk of STEMI was found, whereas higher apoCIII was associated with lower risk of STEMI in the groups divided by quartiles. The highest quartile of apoCIII versus its lowest quartile was associated with an OR of 0.41 (CI 95% 0.24–0.72) for risk of STEMI.

At discharge, 100% of the STEMI patients were on statin therapy. The fast majority was on rosuvastatin 10 mg daily. Values of continuation samples of conventional lipoproteins were available in STEMI patients after 1 year. Mean TC levels were reduced from 5.63 ± 1.09 mmol/L to 3.96 ± 0.73 mmol/L which is a 27% reduction (*p* < 0.001). Mean LDLc was 2.33 ± 0.75 mmol/L compared to 3.48 ± 0.94 at baseline, which is a 27% reduction (*p* < 0.001). HDLc raised from 1.27 ± 0.36 mmol/L to 1.40 ± 0.36 which is an increase of 12% (*p* < 0.001) Median triglyceride level was 1.31 mmol/L (1.00–1.80) at baseline compared to 1.53 mmol/L (1.01–2.22) after 1 year which is a 20% reduction (*p* < 0.001). According to medical record review 7 patients (3.2%) were not on statin therapy after 1 year due to pharmacological side effects.

In the patients with STEMI 83 (38%) events were observed after a mean follow-up duration of 8.94 years. For each baseline lipid and apo species the hazard ratio for MACE was calculated after adjustment for age, gender, and statin use. Neither conventional lipid levels nor apo levels were associated with a recurrent event in the STEMI group (Table 4).

**Table 4:** Risk of MACE according to various levels of lipoproteins and apo proteins in patients with STEMI

			No. at risk	Obs. years	Events	Incidence rate*	(95% CI)	Univariate analysis		Model 1		Model 1-statin users	
								Hazard ratio	(95% CI)	Hazard ratio†	(95% CI)	Hazard ratio#	(95% CI)
Total-c, mmol/L													
High			159	1113	55	4.94	(3.72–6.43)	0.73¯	(0.46–1.15)	0.73¯	(0.45–1.18)	0.75¯	(0.45–1.25)
Quartile		1	45	302	19	6.29	(3.79–9.82)	1	(reference)	1	(reference)	1	(reference)
		2	65	441	23	5.22	(3.31–7.83)	0.80¯	(0.43–1.48)	0.81¯	(0.42–1.53)	0.76¯	(0.38–1.53)
		3	49	341	20	5.87	(3.58–9.06)	0.94¯	(0.50–1.77)	0.99¯	(0.52–1.92)	1.01¯	(0.51-2.00)
		4	61	415	21	5.06	(3.13–7.74)	0.82¯	(0.44–1.53)	0.85¯	(0.44–1.64)	0.82¯	(0.41–1.65)
Per SD increase			1.10					0.99¯	(0.78–1.25)	1.02¯	(0.79–1.30)	1.08¯	(0.83–1.39)
Triglycerides													
High			65	428	25	5.84	(3.78–8.62)	1.10¯	(0.69–1.75)	1.13¯	(0.70–1.81)	1.11¯	(0.68–1.83)
Quartile		1	44	291	18	6.19	(3.67–9.78)	1	(references)	1	(reference)	1	(reference)
		2	34	242	15	6.20	(3.47–10.22)	1.04¯	(0.52–2.09)	1.06¯	(0.53–2.15)	0.93¯	(0.43–1.98)
		3	64	429	22	5.13	(3.21–7.76)	0.89¯	(0.47–1.68)	0.89¯	(0.47–1.70)	0.79¯	(0.40–1.54)
		4	78	538	28	5.20	(3.46–7.52)	0.91¯	(0.50–1.66)	0.94¯	(0.51–1.73)	0.87¯	(0.46–1.64)
Per ln SD increase			0.81					1.01¯	(0.97–1.04)	1.00¯	(0.96–1.04)	1.00¯	(0.96–1.04)
HDLc, mmol/L													
High			159	1118	56	5.01	(3.78–6.50)	0.72¯	(0.46–1.15)	0.70¯	(0.44–1.11)	0.68¯	(0.42–1.12)
Quartile		1	72	462	30	6.49	(4.38–9.27)	1	(references)	1	(reference)	1	(reference)
		2	50	378	14	3.70	(2.02–6.21)	0.56¯	(0.29–1.07)	0.56¯	(0.29–1.08)	0.53¯	(0.27–1.04)
		3	52	309	25	8.09	(5.24–11.94)	1.21¯	(0.71–2.06)	1.18¯	(0.69–2.02)	1.09¯	(0.61–1.93)
		4	46	350	14	4.00	(2.19–6.71)	0.66¯	(0.35–1.24)	0.57¯	(0.29–1.14)	0.60¯	(0.29–1.23)
Per SD increase			0.40					0.95¯	(0.75–1.22)	0.92¯	(0.71–1.20)	0.94¯	(0.71–1.24)
LDLc, mmol/L													
High			156	57	1083	5.26	(3.99–6.92)	0.83¯	(0.52–1.33)	0.90¯	(0.54–1.48)	0.84¯	(0.50–1.41)
Quartile		1	72	295	21	7.12	(4.41–10.88)	1	(references)	1	(reference)	1	(reference)
		2	50	414	20	4.83	(2.95–7.46)	0.68¯	(0.37–1.26)	0.76¯	(0.40–1.45)	0.65¯	(0.33–1.28)
		3	52	491	24	4.89	(3.13–7.27)	0.65¯	(0.36–1.18)	0.69¯	(0.37–1.30)	0.64¯	(0.34–1.23)
		4	46	300	18	6.00	(3.56–9.48)	0.81¯	(0.43–1.52)	0.89¯	(0.46–1.73)	0.77¯	(0.39–1.54)
Per SD increase			1.00					0.96¯	(0.75–1.22)	0.99¯	(0.76–1.28)	0.92¯	(0.75–1.29)
Remnant cholesterol, mmol/L													
High			75 (34)	507	28	5.53	(3.67–7.98)	1.02¯	(0.65–1.61)	1.05	(0.66–1.66)	1.07¯	(0.66–1.73)
		1	44 (20)	291	18	6.19	(3.67–9.78)	1	(reference)	1	(reference)	1	(reference)
		2	33 (15)	232	15	6.47	(3.62–10.66)	1.08¯	(0.54-.2.17)	1.11¯	(0.55–2.25)	0.92¯	(0.43–1.98)
		3	63 (29)	419	22	9.48	(5.94–14.38)	0.91¯	(0.48–1.72)	0.91¯	(0.48–1.74)	0.80¯	(0.41–1.57)
		4	80 (36)	557	28	5.03	(3.34–7.27)	0.88¯	(0.48–1.60)	0.91¯	(0.49–1.67)	0.86¯	(0.46–1.61)
Per SD increase			0.36					1.06¯	(0.95–1.19)	1.04¯	(0.93–1.16)	1.07¯	(0.94–1.21)
Apo A1, g/L													
High			96	651	36	5.53	(3.87–7.66)	1.05¯	(0.68–1.63)	1.01¯	(0.63–1.61)	0.93¯	(0.56–1.55)
Quartile		1	153	1034	61	5.60	(4.51–7.58)	1	(references)	1	(reference)	1	(reference)
		2	30	165	13	7.88	(4.20-13.47)	1.36¯	(0.74–2.47)	1.25¯	(0.67–2.36)	1.11¯	(0.54–2.27)
		3	28	235	6	2.55	(0.94–5.56)	0.48¯	(0.21–1.11)	0.42¯	(0.18–1.02)	0.44¯	(0.18–1.06)
		4	9	65	3	4.62	(0.95–13.49)	0.82¯	(0.29–2.63)	0.70¯	(0.21–2.30)	0.82¯	(0.25–2.72)
Per SD increase			0.31					0.84¯	(0.63–1.13)	0.80¯	(0.59–1.10)	0.78¯	(0.56–1.09)
Apo B, g/L													
High			172	1176	60	5.10	(3.89–6.57)	0.71¯	(0.44–1.16)	0.77¯	(0.46–1.31)	0.67¯	(0.40–1.15)
Quartile		1	17	116	7	6.03	(2.43–12.43)	1	(references)	1	(reference)	1	(reference)
		2	43	275	21	7.64	(4.73–11.67)	1.17 ¯	(0.50–2.78)	1.24¯	(0.48–3.19)	0.97¯	(0.33–2.90)
		3	74	537	24	4.47	(2.86–6.65)	0.75¯	(0.32–1.73)	0.84¯	(0.33–2.15)	0.64¯	(0.22–1.87)
		4	86	571	31	5.43	(3.69–7.71)	0.89¯	(0.39–2.02)	0.99¯	(0.39–2.53)	0.78¯	(0.27–2.23)
Per SD increase			0.29					0.91¯	(0.70–1.19)	0.94¯	(0.71–1.25)	0.93¯	(0.69–1.25)
Apo C-I, mg/L													
Quartile	1		85	544	37	6.80	(4.79–9.37)	1	(references)	1	(reference)	1	(reference)
	2		54	406	17	4.19	(2.44–6.70)	0.65¯	(0.37–1.16)	0.66¯	(0.37–1.18)	0.64¯	(0.34–1.20)
	3		44	294	17	5.78	(3.37–9.26)	0.91¯	(0.51–1.61)	0.89¯	(0.49–1.62)	0.86¯	(0.48–1.60)
	4		37	256	12	4.69	(2.42–8.19)	0.73¯	(0.38–1.40)	0.76¯	(0.39–1.50)	0.73¯	(0.37–1.49)
Per SD increase			5.33					1.03¯	(0.86–1.22)	1.04¯	(0.88–1.22)	1.04¯	(0.88–1.23)
Apo C-II, mg/L													
Quartile	1		47	322	19	5.90	(3.55–9.21)	1	(references)	1	(reference)	1	(reference)
	2		58	379	26	6.86	(4.48–10.05)	1.32¯	(0.68–2.25)	1.34¯	(0.72–2.49)	1.46¯	(0.75–2.86)
	3		52	375	16	4.27	(2.44–6.93)	0.78¯	(0.40–1.52)	0.83¯	(0.42–1.64)	0.89¯	(0.43–1.85)
	4		63	423	22	5.20	(3.26–7.87)	0.96¯	(0.51–1.78)	0.98¯	(0.51–1.86)	1.05¯	(0.52–2.12)
Per ln SD increase			27.64					1.01¯	(0.99–1.02)	1.01¯	(0.99–1.02)	1.01¯	(0.99–1.02)
Apo C-III, mg/L													
Quartile	1		81	509	37	7.27	(5.12–10.02)	1	(references)	1	(reference)	1	(reference)
	2		51	392	14	3.57	(1.95–5.99)	0.55 ¯	(0.30–1.02)	0.55¯	(0.30–1.02)	0.53¯	(0.27–1.03)
	3		40	281	15	5.34	(2.99–8.80)	0.79¯	(0.43–1.44)	0.81¯	(0.44–1.50)	0.75¯	(0.39–1.42)
	4		48	317	17	5.36	(3.12–8.59)	0.81¯	(0.45–1.44)	0.79¯	(0.43–1.44)	0.77¯	(0.41–1.44)
Per SD increase			37.12					1.01¯	(1.00-1.02)	1.01¯	(1.00-1.02)	1.01¯	(1.00-1.02)
Apo E, mg/L													
Quartile	1		45	309	18	5.83	(3.45–9.21)	1	(references)	1	(reference)	1	(reference)
	2		61	367	28	7.63	(5.07–11.03)	1.33¯	(0.73–2.44)	1.49¯	(0.78–2.86)	1.28¯	(0.65–2.53)
	3		62	479	18	3.76	(2.23–5.94)	0.70¯	(0.36–1.36)	0.75¯	(0.38–1.49)	0.81¯	(0.39–1.66)
	4		52	344	19	5.52	(3.33–8.63)	0.99¯	(0.51–1.90)	1.13¯	(0.57–2.26)	0.96¯	(0.46–1.99)
Per SD increase			13.04					1.07¯	(0.98–1.16)	1.07¯	(0.99–1.16)	1.07¯	(0.99–1.16)
Ratio ApoB/ApoA1													
Quartile	1		5	28	4	1.14	(0.31–2.93)	1	(references)	1	(reference)	1	(reference)
	2		27	186	10	5.38	(2.58–9.89)	0.44¯	(0.14–1.40)	0.43¯	(0.13–1.41)	0.55¯	(0.15–2.01)
	3		50	337	20	5.93	(3.63–9.17)	0.46¯	(0.16–1.36)	0.43¯	(0.15–1.33)	0.41¯	(0.12–1.44)
	4		138	948	49	5.17	(3.82–6.83)	0.40¯	(0.14–1.11)	0.41¯	(0.14–1.18)	0.45¯	(0.14–1.48)
Per SD increase			0.25					1.05¯	(0.86–1.27)	1.08¯	(0.88–1.33)	1.07¯	(0.86–1.32)
Non-HDLc, mmol/L													
Quartile	1		33	207	15	7.25	(4.01–11.95)	1	(references)	1	(reference)	1	(reference)
	2		47	341	17	4.99	(2.90–7.98)	0.68¯	(0.34–1.38)	0.70¯	(0.32–1.51)	0.53¯	(0.23–1.12)
	3		85	601	29	4.86	(3.23–6.93)	0.68¯	(0.36–1.27)	0.71¯	(0.37–1.39)	0.65¯	(0.32–1.32)
	4		55	350	22	6.29	(3.94–9.52)	0.87¯	(0.45–1.68)	0.92¯	(0.45–1.89)	0.78¯	(0.38–1.62)
Per SD increase			1.13					1.00¯	(0.78–1.28)	1.05¯	(0.81–1.35)	1.05¯	(0.81–1.37)

Significance: ¯*p* > 0.05

*Model 1; Adjusted for age, sex and statin use

†Model 1-statin users; Adjusted for age and sex, statin users excluded

## Discussion

In this case–control study with 10-year follow-up of the STEMI patients the value of extensive lipid and apo profiling to predict STEMI or MACE was studied. Key findings of the study are: (1) apoA1, apoB and the apoB/apoA1 ratio were strongly associated with the risk of developing a STEMI; (2) remnant cholesterol was significantly associated with risk of STEMI; (3) apoCII, apoCIII and apo E were not clearly associated with risk of STEMI; and (4) no significant association of serum lipids or serum apos with MACE in STEMI patients during follow-up who were all treated with statins during follow-up was found.

Despite current standards of care aimed at achieving targets for LDLc and other traditional risk factors, STEMI patients remain at high risk of new cardiovascular events [8, 13]. The results of this study are in line with this, with a 38% event rate during almost 9-year follow-up.

In recent years, it has been demonstrated that apoA1, apoB and apoB/apoA1 ratio can predict CVD better than LDLc [9–13, 26–28]. In this study similar results were found. Adjusted for age, gender and statin therapy, elevated levels of apoB and apoB/apoA1 ratio were associated with an increased risk of developing a STEMI and LDLc was not. Holmes et al. recently confirmed these results in a nested case–control study showing that apoA1, apoB and apo/apoA1 ratio were strongly associated with risk of MI [15]. Similar results were also obtained in a previous meta-analysis of Sniderman et al. leading to the conclusion that apoB is superior to non-HDLc and that non-HDLc is superior to LDLc as a predictor of CVD risk [16]. Taken all this evidence in mind a slow but progressive shift towards the use of serum apos in clinical practice can be observed. Several position or consensus statements and guidelines from medical associations, therefore, recommend introduction of apoB levels as (secondary) treatment target in clinical practice [29–32].

ApoB concentration represents the total number of atherogenic particles including VLDL, intermediate-density lipoprotein (IDL), IDL remnants, LDL and lipoprotein (a), as each of these particles carries one molecule of apoB. With the routine measurement of apoB, a considerable number of events could be prevented on top of LDLc [16]. The clinical use of apoB levels will be an important step towards precision medicine. It would be ideal if—in the future—events of an individual patient may be predicted and hereby improve risk stratification for future events. For example, recently Hermans et al. showed that premature CAD may occur in patients with an apoCII deficiency with normotriglyceridemia [33]. Despite the fact that these patients had a low a priori risk for CAD [54], they presented with STEMI at young age and had a high relative risk of 10-year re-infarction or revascularization [30]. This contrasts with the phenotype described in textbooks, where a total lack of apoCII is assumed to result in intravascular TG accumulation because of inactivation of LPL, whereby delayed intravascular TG lipolysis is a strong and independent predictor of CAD [34, 35].

So far, only serum apo A1 and B were measured in medical laboratories, whereas the apos CI, CII, CIII and E are not routinely measured. To determine the full panel of serum apos in a multiplexed and immunoassay independent way, van den Broek et al. [19] recently developed a quantitative serum apo profiling test using LC–MS/MS. This LC–MS/MS test produces highly accurate test results, which are in concordance with quality requirements for medical tests. Furthermore, the test is not confounded by hypertriglyceridemia [19]. Moreover, the multiplex apo test can be performed in the non-fasting state [25].

Remnant cholesterol is the cholesterol content of triglyceride-rich lipoproteins, composed of mainly VLDL and IDL [36, 37]. The current study shows that remnant cholesterol level was associated with an increased risk of STEMI. These results are in concordance with the results of several other studies [36, 38]. Varbo et al. implied a causal risk of elevated remnant cholesterol levels for ischemic heart disease, independent of HDLc levels [38].

The VLDL-associated apos apoCII, apoE and—to a large extent—apoCIII have recently been identified as potentially important new risk factors [17, 18]. These apos are abundant on TG-rich lipoproteins, strongly modulate their metabolism [39], and might play an important role in the development of atherosclerosis and subsequent CVD.

Recently, Pechlaner et al. [17] showed that apoCII, apoCIII and apoE were strongly associated with incident CVD in the general community. This finding was supported by Van Capelleveen et al. [18]. These results could, however, not be confirmed in the current case–control study. In fact, no clear relation was found between VLDL-associated apos and the risk of STEMI. These conflicting results could have several explanations. First of all, the blood samples of the cases were drawn soon after admission to the hospital, i.e., in a non-fasting state. The blood samples of the control group were obtained in a fasting state. Second, in both cases and controls the TG levels are 0.5 mmol/L lower than in the EPIC Norfolk population study, so the results in the current study may not be generalizable with the EPIC Norfolk population study. Third, the information about the percentage of controls that have diabetes mellitus (DM) was self-reported (1.7%). In the STEMI group 7.7% had DM.

The relation of apoE with CVD risk is more controversial. Although Pechlaner et al. found a strong association, a meta-analysis with almost 10,000 individuals and 1400 events recently performed by Sofat et al. found no association of apoE with CVD [40].

In STEMI patients, no association of baseline serum lipids or serum apos with MACE during long-term follow-up was found. Although earlier results from the TNT study [41] suggest that baseline apoB and apoA1 levels are associated with residual risk in a statin-treated secondary prevention population, the current results did not confirm these findings. This can be due to several reasons. First of all, 100% of the patients were put on statin therapy after their STEMI which clearly had an effect on baseline lipid and apo concentrations which could diminish the relation with cardiovascular events. It has been demonstrated that statin therapy reduces 5-year incidence of major coronary events by about 20% per mmol/L reduction in LDL cholesterol [42]. In this cohort a 27% percentage reduction in LDL-c after 1 year was observed. This significant reduction in lipid concentrations together with the limited number of recurrent events could explain why no association was found between apos or lipid concentrations and recurrent events. Unfortunately, all measured apos were only available as baseline samples and not as continuation samples, so the impact of statin therapy on apo levels in this cohort is unknown. For example, van Lennep et al. demonstrated that, in patients with effective statin treatment, on-treatment levels of apoB and apoA1 were significantly predictive for recurrent events in CAD patients [43]. Second, the determinants of residual risk in statin-treated patients are multifactorial. For example. smoking, hypertension, diabetes, high BMI and higher inflammation grades all contribute to a higher residual risk [15, 41, 44, 45]. All these (modifiable) risk factors could have played a role in the occurrence of a recurrent event and perhaps did these risk factors obscure the association of apos and lipids with recurrent events. At last, the mean follow-up duration was almost 9 years, instead of 5 years in the TNT study.

The primary goal in STEMI patients is to reduce the residual risk as much as possible. Statin therapy has shown to reduce the residual risk substantially by reducing LDLc levels, [46] but the effect on LDLc levels is confined, [47, 48] so the need for other powerful cholesterol-lowering agents or other modifiable risk factors is needed. The IMPROVE-IT study showed that ezetimibe provides an incremental reduction in LDLc of 15–20% which resulted in an increased risk reduction for cardiovascular events [49]. Furthermore, recently, the Fourier study demonstrated their results with the PCSK9 antagonist evolucumab. They showed an additional lowering of more than 50% of LDLc levels on top of statin therapy with evolucumab compared to placebo in high-risk patients. Inhibition of PCSK-9 reduced furthermore the risk of cardiovascular events [50]. In the nearby future the results from the ODDESSEY OUTCOME will be published [51], where 18,000 post ACS patients were administrated with either alirocumab or placebo. Preliminary results showed us that these powerfull cholesterol-lowering agents further reduced residual cardiovascular risk by further lowering LDL-c levels. However, as we currently know, LDLc is not discriminating and refined enough to identify high-risk patients and we should need to work towards better, more meaningfull and well characterized medical tests such as apos to measure pharmacological effects. Current AHA and ESC dyslipidemia guidelines mainly focus on LDLc reduction as primary treatment target. However, in line with several position statements, guidelines and the INTERHEART study, [13, 29–31] valuable effort should be made to substantially modify apoB and apoB/apoA1 ratio. These apos can reliably be measured and a substantial modification could lead to further reduction of the residual cardiovascular risk.

Some potential limitations deserve a comment. First, the controls of the MEGA population were < 70 years; so, results from the case–control study only apply to individuals < 70 years. Second, apoC and E levels were measured in MEGA controls in serum that was thawed twice. However, Bland–Altman plots and scatterplots suggest that this did not lead to measurement error as apoA1 and apoB levels that were measured on both once and twice thawed serum showed equivalent levels with *r*^2^ of 0.89 and 0.93, respectively, but the effect of thawing on apoC and apoE levels is unknown. Furthermore, the models for risk of STEMI and MACE were adjusted for age, gender and statin use. An important factor modifying the association between (apo) lipoproteins and outcome is inflammation. Since C-reactive protein was measured in the acute phase in the STEMI cohort, and in the non-acute phase in the MEGA cohort, we were not able to reliably adjust the apo (lipoproteins) for inflammation.

## Conclusion

In conclusion, apoA1, apoB, and apoB/apoA1 ratio and remnant cholesterol were strongly associated with risk of STEMI, the apoB/apoA1 ratio being superior to LDLc and non-HDLc. Second, no clear relation of apoCI, apoCII, apoCIII and apoE with the risk of STEMI as compared with a population-based control group was found. Neither serum lipids nor serum apos predicted death, re-infarction or revascularization in statin-treated patients during follow-up after a first STEMI. Valuable effort should be made to further reduce residual cardiovascular risk by intensive life style modification, by testing new powerful cholesterol-lowering agents and using additional more discriminating and more refined treatments targets like apoB and apoB/apoA1 ratio.

## References

[1] Randomised trial of cholesterol lowering (1994). in 4444 patients with coronary heart disease: the Scandinavian Simvastatin Survival Study (4S). Lancet.

[2] Sacks FM, Pfeffer MA, Moye LA, Rouleau JL, Rutherford JD, Cole TG, Brown L, Warnica JW, Arnold JM, Wun CC, Davis BR, Braunwald E (1996). The effect of pravastatin on coronary events after myocardial infarction in patients with average cholesterol levels. Cholesterol and Recurrent Events Trial investigators. N Engl J Med.

[3] Shepherd J, Cobbe SM, Ford I, Isles CG, Lorimer AR, MacFarlane PW, McKillop JH, Packard CJ (1995). Prevention of coronary heart disease with pravastatin in men with hypercholesterolemia. West of Scotland Coronary Prevention Study Group. N Engl J Med.

[4] Marz W, Kleber ME, Scharnagl H, Speer T, Zewinger S, Ritsch A, Parhofer KG, von Eckardstein A, Landmesser U, Laufs U (2017). HDL cholesterol: reappraisal of its clinical relevance. Clin Res Cardiol.

[5] Zethelius B, Berglund L, Sundstrom J, Ingelsson E, Basu S, Larsson A, Venge P, Arnlov J (2008). Use of multiple biomarkers to improve the prediction of death from cardiovascular causes. N Engl J Med.

[6] Libby P (2005). The forgotten majority: unfinished business in cardiovascular risk reduction. J Am Coll Cardiol.

[7] Wong ND, Zhao Y, Quek RGW, Blumenthal RS, Budoff MJ, Cushman M, Garg P, Sandfort V, Tsai M, Lopez JAG (2017). Residual atherosclerotic cardiovascular disease risk in statin-treated adults: the multi-ethnic study of atherosclerosis. J Clin Lipidol.

[8] Fruchart JC, Sacks FM, Hermans MP, Assmann G, Brown WV, Ceska R, Chapman MJ, Dodson PM, Fioretto P, Ginsberg HN, Kadowaki T, Lablanche JM, Marx N, Plutzky J, Reiner Z, Rosenson RS, Staels B, Stock JK, Sy R, Wanner C, Zambon A, Zimmet P (2008). The Residual Risk Reduction Initiative: a call to action to reduce residual vascular risk in dyslipidaemic patient. Diab Vasc Dis Res.

[9] Walldius G, Jungner I, Holme I, Aastveit AH, Kolar W, Steiner E (2001). High apolipoprotein B, low apolipoprotein A-I, and improvement in the prediction of fatal myocardial infarction (AMORIS study): a prospective study. Lancet.

[10] Lamarche B, Moorjani S, Lupien PJ, Cantin B, Bernard PM, Dagenais GR, Despres JP (1996). Apolipoprotein A-I and B levels and the risk of ischemic heart disease during a five-year follow-up of men in the Quebec cardiovascular study. Circulation.

[11] Intervention The Long-term, with Pravastatin in Ischaemic Disease (LIPID) study group (1998). Prevention of cardiovascular events and death with pravastatin in patients with coronary heart disease and a broad range of initial cholesterol levels. N Engl J Med.

[12] Sniderman AD, Islam S, McQueen M, Pencina M, Furberg CD, Thanassoulis G, Yusuf S (2016). Age and cardiovascular risk attributable to apolipoprotein B, low-density lipoprotein cholesterol or non-high-density lipoprotein cholesterol. J Am Heart Assoc.

[13] McQueen MJ, Hawken S, Wang X, Ounpuu S, Sniderman A, Probstfield J, Steyn K, Sanderson JE, Hasani M, Volkova E, Kazmi K, Yusuf S (2008). Lipids, lipoproteins, and apolipoproteins as risk markers of myocardial infarction in 52 countries (the INTERHEART study): a case-control study. Lancet.

[14] Pischon T, Girman CJ, Sacks FM, Rifai N, Stampfer MJ, Rimm EB (2005). Non-high-density lipoprotein cholesterol and apolipoprotein B in the prediction of coronary heart disease in men. Circulation.

[15] Holmes MV, Millwood IY, Kartsonaki C, Hill MR, Bennett DA, Boxall R, Guo Y, Xu X, Bian Z, Hu R, Walters RG, Chen J, Ala-Korpela M, Parish S, Clarke RJ, Peto R, Collins R, Li L, Chen Z (2018). Lipids, lipoproteins, and metabolites and risk of myocardial infarction and stroke. J Am Coll Cardiol.

[16] Sniderman AD, Williams K, Contois JH, Monroe HM, McQueen MJ, de Graaf J, Furberg CD (2011). A meta-analysis of low-density lipoprotein cholesterol, non-high-density lipoprotein cholesterol, and apolipoprotein B as markers of cardiovascular risk. Circ Cardiovasc Qual Outcomes.

[17] Pechlaner R, Tsimikas S, Yin X, Willeit P, Baig F, Santer P, Oberhollenzer F, Egger G, Witztum JL, Alexander VJ, Willeit J, Kiechl S, Mayr M (2017). Very-low-density lipoprotein-associated apolipoproteins predict cardiovascular events and are lowered by inhibition of APOC-III. J Am Coll Cardiol.

[18] van Capelleveen JC, Moens Bernelot SJ, Yang X, Kastelein JJP, Wareham NJ, Zwinderman AH, Stroes ESG, Witztum JL, Hovingh GK, Khaw KT, Boekholdt SM, Tsimikas S (2017). Apolipoprotein C-III levels and incident coronary artery disease risk: the EPIC-Norfolk prospective population study. Arterioscler Thromb Vasc Biol.

[19] van den Broek I, Romijn FP, Nouta J, van der Laarse A, Drijfhout JW, Smit NP (2016). Automated Multiplex LC-MS/MS Assay for Quantifying Serum Apolipoproteins A-I, B, C-I, C-II, C-III, and E with Qualitative Apolipoprotein E Phenotyping. Clin chem.

[20] van der Hoeven BL, Liem SS, Jukema JW, Suraphakdee N, Putter H, Dijkstra J, Atsma DE, Bootsma M, Zeppenfeld K, Oemrawsingh PV, van der Wall EE, Schalij MJ (2008). Sirolimus-eluting stents versus bare-metal stents in patients with ST-segment elevation myocardial infarction: 9-month angiographic and intravascular ultrasound results and 12-month clinical outcome results from the MISSION! Intervention Study. J Am Coll Cardiol.

[21] van Stralen KJ, Rosendaal FR, Doggen CJ (2008). Minor injuries as a risk factor for venous thrombosis. Arch Intern Med.

[22] Liem SS, van der Hoeven BL, Oemrawsingh PV, Bax JJ, van der Bom JG, Bosch J, Viergever EP, van Rees C, Padmos I, Sedney MI, van Exel HJ, Verwey HF, Atsma DE, van der Velde ET, Jukema JW, van der Wall EE, Schalij MJ (2007). MISSION!: optimization of acute and chronic care for patients with acute myocardial infarction. Am Heart J.

[23] O’Gara PT, Kushner FG, Ascheim DD, Casey DE, Chung MK, de Lemos JA, Ettinger SM, Fang JC, Fesmire FM, Franklin BA, Granger CB, Krumholz HM, Linderbaum JA, Morrow DA, Newby LK, Ornato JP, Ou N, Radford MJ, Tamis-Holland JE, Tommaso CL, Tracy CM, Woo YJ, Zhao DX, Anderson JL, Jacobs AK, Halperin JL, Albert NM, Brindis RG, Creager MA, DeMets D, Guyton RA, Hochman JS, Kovacs RJ, Kushner FG, Ohman EM, Stevenson WG, Yancy CW (2013). ACCF/AHA guideline for the management of ST-elevation myocardial infarction: a Report of the American College of Cardiology Foundation/American Heart Association Task Force on Practice Guidelines. J Am Coll Cardiol.

[24] Steg PG, James SK, Atar D, Badano LP, Blomstrom-Lundqvist C, Borger MA, Di Mario C, Dickstein K, Ducrocq G, Fernandez-Aviles F, Gershlick AH, Giannuzzi P, Halvorsen S, Huber K, Juni P, Kastrati A, Knuuti J, Lenzen MJ, Mahaffey KW, Valgimigli M, van ‘t Hof A, Widimsky P, Zahger D (2012). ESC Guidelines for the management of acute myocardial infarction in patients presenting with ST-segment elevation. Eur Heart J.

[25] Nordestgaard BG, Langsted A, Mora S, Kolovou G, Baum H, Bruckert E, Watts GF, Sypniewska G, Wiklund O, Boren J, Chapman MJ, Cobbaert C, Descamps OS, von Eckardstein A, Kamstrup PR, Pulkki K, Kronenberg F, Remaley AT, Rifai N, Ros E, Langlois M (2016). Fasting is not routinely required for determination of a lipid profile: clinical and laboratory implications including flagging at desirable concentration cutpoints—a joint consensus statement from the European Atherosclerosis Society and European Federation of Clinical Chemistry and Laboratory Medicine. Clin Chem.

[26] Kappelle PJ, Gansevoort RT, Hillege JL, Wolffenbuttel BH, Dullaart RP (2011). Apolipoprotein B/A-I and total cholesterol/high-density lipoprotein cholesterol ratios both predict cardiovascular events in the general population independently of nonlipid risk factors, albuminuria and C-reactive protein. J Intern Med.

[27] Pencina MJ, D’Agostino RB, Zdrojewski T, Williams K, Thanassoulis G, Furberg CD, Peterson ED, Vasan RS, Sniderman AD (2015). Apolipoprotein B improves risk assessment of future coronary heart disease in the Framingham Heart Study beyond LDL-C and non-HDL-C. Eur J Prev Cardiol.

[28] Thanassoulis G, Williams K, Ye K, Brook R, Couture P, Lawler PR, de Graaf J, Furberg CD, Sniderman A (2014). Relations of change in plasma levels of LDL-C, non-HDL-C and apoB with risk reduction from statin therapy: a meta-analysis of randomized trials. J Am Heart Assoc.

[29] Contois JH, McConnell JP, Sethi AA, Csako G, Devaraj S, Hoefner DM, Warnick GR (2009). Apolipoprotein B and cardiovascular disease risk: position statement from the AACC Lipoproteins and Vascular Diseases Division Working Group on Best Practices. Clin Chem.

[30] Brunzell JD, Davidson M, Furberg CD, Goldberg RB, Howard BV, Stein JH, Witztum JL (2008). Lipoprotein management in patients with cardiometabolic risk: consensus conference report from the American Diabetes Association and the American College of Cardiology Foundation. J Am Coll Cardiol.

[31] Jellinger PS, Handelsman Y, Rosenblit PD, Bloomgarden ZT, Fonseca VA, Garber AJ, Grunberger G, Guerin CK, Bell DSH, Mechanick JI, Pessah-Pollack R, Wyne K, Smith D, Brinton EA, Fazio S, Davidson M (2017). American association of clinical endocrinologists and american college of endocrinology guidelines for management of dyslipidemia and prevention of cardiovascular disease. Endocr Pract.

[32] Langlois MR, Cobbaert CMJ, Mora C, Remaley S, Ros AT, Watts E, Borén GF, Baum J, Bruckert H, Catapano E, Descamps A, von Eckardstein OS, Kamstrup A, Kolovou PR, Kronenberg G, Langsted F, Pulkki A, Rifai K, Sypniewska N, Wiklund GO, Nordestgaard BG, for the European Atherosclerosis Society (EAS) and the European Federation of Clinical Chemistry and Laboratory Medicine (EFLM) joint Consensus Initiative (2018). Quantifying atherogenic lipoproteins: current and future challenges in the era of personalized medicine and very low LDL-cholesterol. A consensus statement from EAS and EFLM. Clin Chem.

[33] Hermans MPJ, Bodde MC, Jukema JW, Schalij MJ, van der Laarse A, Cobbaert CM (2017). Low levels of apolipoprotein-CII in normotriglyceridemic patients with very premature coronary artery disease: observations from the MISSION! Intervention study. J Clin Lipidol.

[34] Sposito AC, Lemos PA, Santos RD, Hueb W, Vinagre CG, Quintella E, Carneiro O, Chapman MJ, Ramires JA, Maranhao RC (2004). Impaired intravascular triglyceride lipolysis constitutes a marker of clinical outcome in patients with stable angina undergoing secondary prevention treatment: a long-term follow-up study. J Am Coll Cardiol.

[35] Fukushima H, Sugiyama S, Honda O, Koide S, Nakamura S, Sakamoto T, Yoshimura M, Ogawa H, Fujioka D, Kugiyama K (2004). Prognostic value of remnant-like lipoprotein particle levels in patients with coronary artery disease and type II diabetes mellitus. J Am Coll Cardiol.

[36] Chapman MJ, Ginsberg HN, Amarenco P, Andreotti F, Boren J, Catapano AL, Descamps OS, Fisher E, Kovanen PT, Kuivenhoven JA, Lesnik P, Masana L, Nordestgaard BG, Ray KK, Reiner Z, Taskinen MR, Tokgozoglu L, Tybjaerg-Hansen A, Watts GF (2011). Triglyceride-rich lipoproteins and high-density lipoprotein cholesterol in patients at high risk of cardiovascular disease: evidence and guidance for management. Eur Heart J.

[37] Nordestgaard BG, Benn M, Schnohr P, Tybjaerg-Hansen A (2007). Nonfasting triglycerides and risk of myocardial infarction, ischemic heart disease, and death in men and women. JAMA.

[38] Varbo A, Benn M, Tybjaerg-Hansen A, Jorgensen AB, Frikke-Schmidt R, Nordestgaard BG (2013). Remnant cholesterol as a causal risk factor for ischemic heart disease. J Am Coll Cardiol.

[39] Jong MC, Hofker MH, Havekes LM (1999). Role of ApoCs in lipoprotein metabolism: functional differences between ApoC1, ApoC2, and ApoC3. Arterioscler Thromb Vasc Biol.

[40] Sofat R, Cooper JA, Kumari M, Casas JP, Mitchell JP, Acharya J, Thom S, Hughes AD, Humphries SE, Hingorani AD (2016). Circulating apolipoprotein E concentration and cardiovascular disease risk: meta-analysis of results from three studies. PLoS Med.

[41] Mora S, Wenger NK, Demicco DA, Breazna A, Boekholdt SM, Arsenault BJ, Deedwania P, Kastelein JJ, Waters DD (2012). Determinants of residual risk in secondary prevention patients treated with high- versus low-dose statin therapy: the treating to new targets (TNT) study. Circulation.

[42] Baigent C, Keech A, Kearney PM, Blackwell L, Buck G, Pollicino C, Kirby A, Sourjina T, Peto R, Collins R, Simes R (2005). Efficacy and safety of cholesterol-lowering treatment: prospective meta-analysis of data from 90,056 participants in 14 randomised trials of statins. Lancet.

[43] van Lennep JE, Westerveld HT, van Lennep HW, Zwinderman AH, Erkelens DW, van der Wall EE (2000). Apolipoprotein concentrations during treatment and recurrent coronary artery disease events. Arterioscler Thromb Vasc Biol.

[44] Ridker PM, Everett BM, Thuren T, MacFadyen JG, Chang WH, Ballantyne C, Fonseca F, Nicolau J, Koenig W, Anker SD, Kastelein JJP, Cornel JH, Pais P, Pella D, Genest J, Cifkova R, Lorenzatti A, Forster T, Kobalava Z, Vida-Simiti L, Flather M, Shimokawa H, Ogawa H, Dellborg M, Rossi PRF, Troquay RPT, Libby P, Glynn RJ (2017). Antiinflammatory therapy with canakinumab for atherosclerotic disease. N Engl J Med.

[45] Kulenthiran S, Ewen S, Bohm M, Mahfoud F (2017). Hypertension up to date: SPRINT to SPYRAL. Clin Res Cardiol.

[46] Baigent C, Blackwell L, Emberson J, Holland LE, Reith C, Bhala N, Peto R, Barnes EH, Keech A, Simes J, Collins R (2010). Efficacy and safety of more intensive lowering of LDL cholesterol: a meta-analysis of data from 170,000 participants in 26 randomised trials. Lancet.

[47] Weng TC, Yang YH, Lin SJ, Tai SH (2010). A systematic review and meta-analysis on the therapeutic equivalence of statins. J Clin Pharm Ther.

[48] Laufs U, Karmann B, Pittrow D (2016). Atorvastatin treatment and LDL cholesterol target attainment in patients at very high cardiovascular risk. Clin Res Cardiol.

[49] Cannon CP, Blazing MA, Giugliano RP, McCagg A, White JA, Theroux P, Darius H, Lewis BS, Ophuis TO, Jukema JW, De Ferrari GM, Ruzyllo W, De Lucca P, Im K, Bohula EA, Reist C, Wiviott SD, Tershakovec AM, Musliner TA, Braunwald E, Califf RM (2015). Ezetimibe added to statin therapy after acute coronary syndromes. N Engl J Med.

[50] Sabatine MS, Giugliano RP, Keech AC, Honarpour N, Wiviott SD, Murphy SA, Kuder JF, Wang H, Liu T, Wasserman SM, Sever PS, Pedersen TR (2017). Evolocumab and clinical outcomes in patients with cardiovascular disease. N Engl J Med.

[51] Schwartz GG, Bessac L, Berdan LG, Bhatt DL, Bittner V, Diaz R, Goodman SG, Hanotin C, Harrington RA, Jukema JW, Mahaffey KW, Moryusef A, Pordy R, Roe MT, Rorick T, Sasiela WJ, Shirodaria C, Szarek M, Tamby JF, Tricoci P, White H, Zeiher A, Steg PG (2014). Effect of alirocumab, a monoclonal antibody to PCSK9, on long-term cardiovascular outcomes following acute coronary syndromes: rationale and design of the ODYSSEY outcomes trial. Am Heart J.

[52] Catapano AL, Graham I, De Backer G, Wiklund O, Chapman MJ, Drexel H, Hoes AW, Jennings CS, Landmesser U, Pedersen TR, Reiner Z, Riccardi G, Taskinen M-R, Tokgozoglu L, Verschuren WMM, Vlachopoulos C, Wood DA, Zamorano JL (2016). 2016 ESC/EAS Guidelines for the Management of Dyslipidaemias. Eur Heart J.

[53] Stone NJ, Robinson JG, Lichtenstein AH, Bairey Merz CN, Blum CB, Eckel RH, Goldberg AC, Gordon D, Levy D, Lloyd-Jones DM, McBride P, Schwartz JS, Shero ST, Smith SC, Watson K, Wilson PWF (2014). 2013 ACC/AHA guideline on the treatment of blood cholesterol to reduce atherosclerotic cardiovascular risk in adults. Circulation.

[54] Wilson PWF, D’Agostino RB, Levy D, Belanger AM, Silbershatz H, Kannel WB (1998). Prediction of coronary heart disease using risk factor categories. Circulation.

